# Retraction: Observations on the efficacy of edaravone dexborneol in preventing post-stroke depression and its inflammatory mechanism: a prospective, randomized, control trial

**DOI:** 10.3389/fnins.2026.1860508

**Published:** 2026-04-28

**Authors:** Frontiers Editorial Office

The journal retracts the September 09 2024 article cited above.

Following publication, concerns were raised regarding the Clinical Trial Registration Number associated with the article. An investigation was conducted in accordance with Frontiers' policies.

The investigation found that the clinical trial registration number provided by the authors did not cover the experiments reported in the article. The authors were unable to provide official documentation showing that these experiments were prospectively documented and approved, or to provide a satisfactory explanation in response to these concerns. As a result, the article was found to be in breach of Frontiers' research ethics policies and has therefore been retracted.

This retraction was approved by the Chief Editors of Frontiers in Neuroscience and the Chief Executive Editor of Frontiers. The authors were informed of this decision and given the opportunity to respond. A record of this communication is held by the publisher.

Frontiers would like to thank Kareem Hasan for contacting the journal regarding the published article.

